# Head rice rate measurement based on concave point matching

**DOI:** 10.1038/srep41353

**Published:** 2017-01-27

**Authors:** Yuan Yao, Wei Wu, Tianle Yang, Tao Liu, Wen Chen, Chen Chen, Rui Li, Tong Zhou, Chengming Sun, Yue Zhou, Xinlu Li

**Affiliations:** 1 Jiangsu Key Laboratory of Crop Genetics and Physiology/Co-Innovation Center for Modern Production Technology of Grain Crops, Yangzhou University, Yangzhou 225009, China

## Abstract

Head rice rate is an important factor affecting rice quality. In this study, an inflection point detection-based technology was applied to measure the head rice rate by combining a vibrator and a conveyor belt for bulk grain image acquisition. The edge center mode proportion method (ECMP) was applied for concave points matching in which concave matching and separation was performed with collaborative constraint conditions followed by rice length calculation with a minimum enclosing rectangle (MER) to identify the head rice. Finally, the head rice rate was calculated using the sum area of head rice to the overall coverage of rice. Results showed that bulk grain image acquisition can be realized with test equipment, and the accuracy rate of separation of both indica rice and japonica rice exceeded 95%. An increase in the number of rice did not significantly affect ECMP and MER. High accuracy can be ensured with MER to calculate head rice rate by narrowing down its relative error between real values less than 3%. The test results show that the method is reliable as a reference for head rice rate calculation studies.

According to the Chinese national standard “GB/T 21719–2008”, head rice is milled rice kernels that are three-fourths of the original kernel length after complete milling. Head rice ratio refers to the mass ratio of head rice to the overall rice sample, which significantly affects rice quality, and is considered an important requirement in the purchasing of rice. Therefore, a more automated detection of head rice yield is of practical use.

The key to detecting head rice yield is to accurately quantify the number of head rice. The present head rice rate detection method is performed by screen method^1^, which is time consuming, unreliable and easily affected by human factors^2^,^3^. It is far from the require of the rapid, accurate, and objective detection of rice quality desired by the grain trade.

Computer vision technology has been increasingly widely used in agriculture^4^,^5^,^6^ and the study of rice quality, including geometric characteristics of grain^7^,^8^,^9^, crack analysis^10^,^11^,^12^,^13^, analysis of chalkiness^14^,^15^, and transparency analysis^16^. Some progress has shown in head rice yield detection using computer vision technology. Yadav *et al*.^17^ have established a quantitative estimation model of head milled rice through extracting characteristics including length, perimeter, and area of head rice and broken rice and found the minimum RMSE to be 1.1%. Van^18^ detected head and broken rice with platform scanning and image analysis technology, the results suggested that efficiency significantly increased over manual calculation while accuracy was maintained. However, detection equipment for head rice has not been systematically described yet, including bulk construction of such equipment and description of an efficient detection method of milled rice.

In this study, rice adhesion was separated based on the batch-obtained image of rice using a concave point detection method to calculate grain length and head rice yield. Although the national standard for the milled rice rate detection method is calculated according to the mass ratio, area ratio is usually used to replace mass ratio in computer vision technology^19^. Therefore, we calculated the head rice yield as the ratio of head rice to the overall grain pixel number, which confirmed in this study, is a fast, repeatable and accurate detection method which provides a foundation for the acquisition of head rice yield by computer vision technology.

## Materials and Methods

### Experimental equipment and process

**Figure 1:**
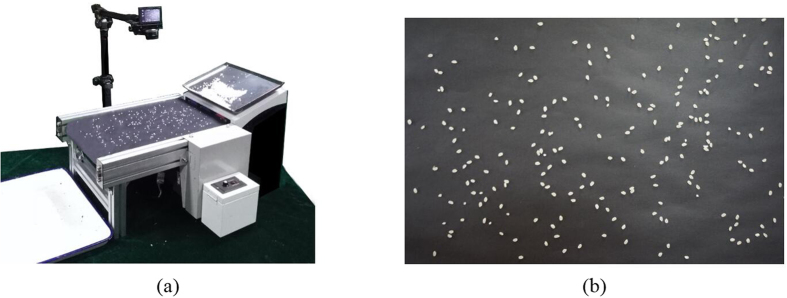
Image acquisition device and raw image.

To explore the adaptability of this method to different types of rice grains, two representative varieties, Lianjing 7 (*Japonica*) and Yangliangyou 6 (*Indica*) were selected and hulled using a hulling mill (SY88-TH, Yongzhou, China; W340 × L660 × H720, 220 V/0.4 KW) and milled with an experimental semi-automatic milling machine (SY2001-NSART100, South Korea; W330 × L500 × H740, 220 V/0.4 KW). Imaging acquisition equipment, presented in Fig. 1(a), was composed of vibrator (voltage, 220 V; frequency, 20 Hz), belt (length, 30 cm; width, 25 cm; voltage, 220 V; speed, 60 mm/s) and digital camera (NEX-5R; Sony, Japan). Windows 7 64 operating system was used for the computer configuration a, Xeon E3-1230 v3 processor, and a Kingston 8 G memory. Matlab R2014b (The Math Works, Natick, USA) was used as the image processing tool.

**Figure 2:**
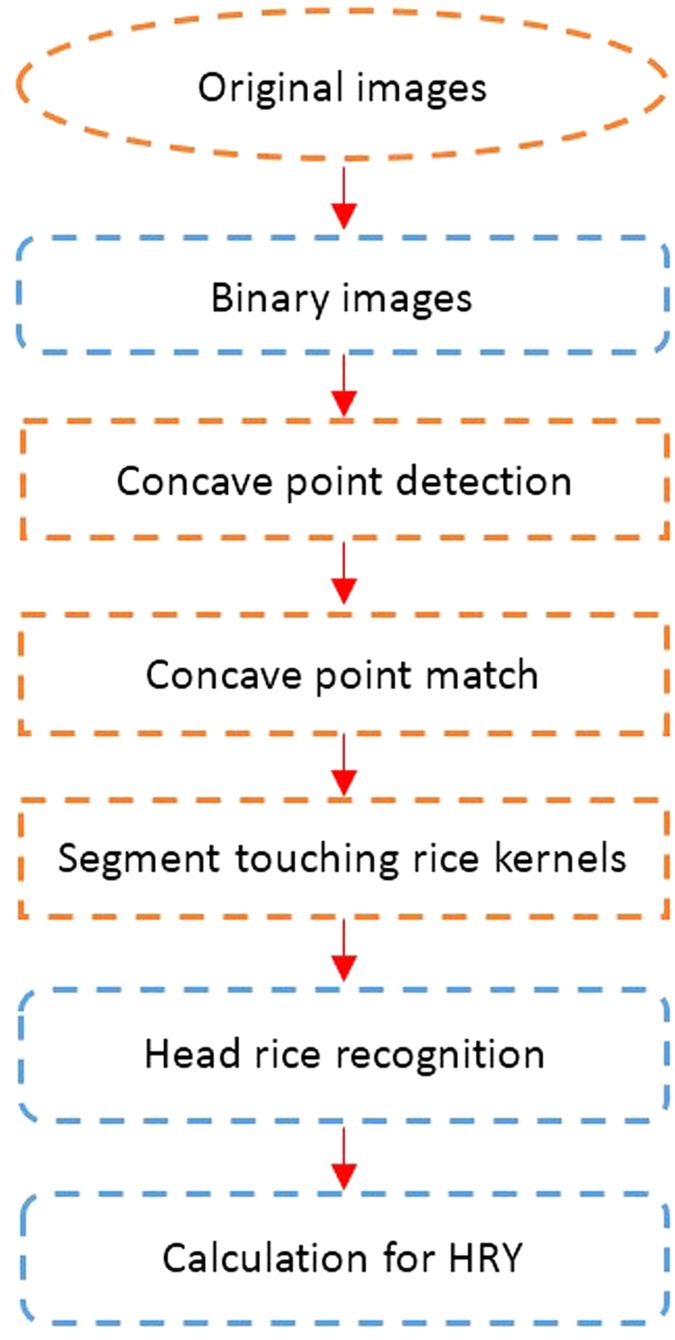
Calculation of head rice yield.

A belt with a black ground with a vibrating oscillator was used as the flow loading platform in the study. The grains fell evenly on the conveyor belt. The camera was placed 30 cm vertically above the transmission belt. With the belt rotating at a constant speed, an image was taken every 5 s. The captured images are shown in Fig. 1(b). The process of detecting head rice by image acquisition is presented in Fig. 2.

### Image preprocessing

**Figure 3:**
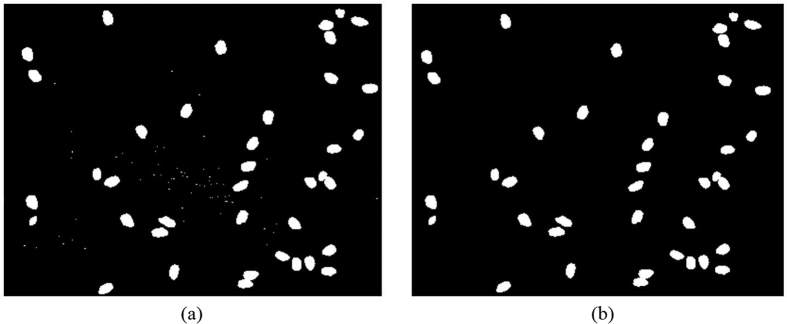
Extraction of grain image.

In image preprocessing, grains were extracted from the background followed by noise removal and edge smoothing. A black belt was used in the experiment, and the grains was extracted using an adaptive threshold segmentation method^20^. The black belt produced a slight noise reflection (Fig. 3(a)). There are several different denoising algorithms^10^,^21^ including mean filtering algorithm, median filter algorithm and wiener filtering algorithm. Different denoising algorithms have different effect on different noises. However, the edge of grain and calculation of grain length could be affected by these algorithms. Hence, all the connecting areas in the image were marked; that is, those in which the pixel number of noise was significantly lower than that of the grains, meaning that the noise can be removed by setting appropriate thresholds. The edge pixel of rice would not be affected. Finally, a 3 × 3 pixel template was used for median filtering to smooth the grain edge^22^. Results are shown in Fig. 3(b).

### Concave point detection

With the coordination of the vibrator and the conveyor belt, most grains were spread out with little touch. By analyzing the features of the touching grains, it was found that two concave points were formed at the point of touch. Therefore, grains can be separated by connecting the matched concave points, suggesting that detection and matching of the concave points are the key points of the separation of the touching grains.

In order to detect the concave points, the edge center mode proportion method (ECMP) was proposed. Specifically, an appropriate template was selected and the pixel of the grain with minimum coordinates as the center to calculate the share of pixels representing grains clockwise. The value of ECMP can be used to represent the concavity of the rice edge, with a higher ECMP indicating a more convex edge and a lower ECMP indicating a more concave edge. The equation used to calculate ECMP is:


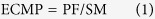


where PF is the pixels of the foreground and SM is the size of the mode.

**Figure 4:**
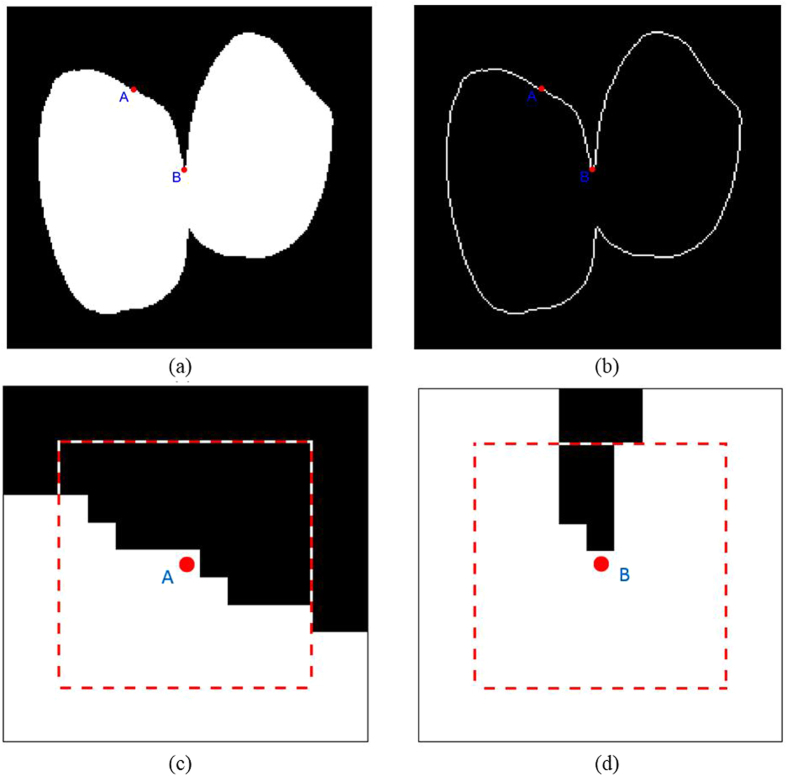
Algorithm of inflection point determination. Note: In (**a**), point A is the pixel of the edge of normal grains; point B is the concave point of its touch with other grain. (**b**) Shows the extracted outline of a grain. (**c**) Shows the schematic diagram of the template centered with point A on edge of a normal grain, and the area outlined with a dashed line is a 9 × 9 template. (**d**) Shows the schematic diagram centered with concave point B.

As indicated in Fig. 4(a), in order to calculate the ECMP of each pixel located on the edge of the grain, a Sobel operator was used for edge detection of grain^23^, as indicated in Fig. 4(b). The template selection depends on the image size with a scale of approximately 300:1. Images with pixels of 3000 × 2500 were used in this experiment, and the template was 9 × 9 pixels. Figure 4(c) and (d) showed the concave-convex degree at different locations of the grains. The ECMP of all grain edges was calculated according to eq. (1), and a figure was drawn with edge points along the horizontal axis and the ECMP values along the vertical axis. Let 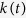 represent the curvilinear function, then the concave region can be expressed as:


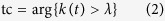


where 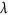 is the threshold segmenting the concave points from the other points. The equation^24^ for λ is:


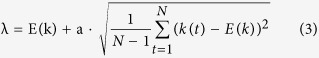


where N is the number of sample points, E(k) is the mean value of k(t), a is the coefficient of the variety. The value of a is smaller for varieties with a long length. Typically, the value of japonica rice is set to approximately 3 and 2 for indica rice, respectively.

### Concave point matching

**Figure 5:**
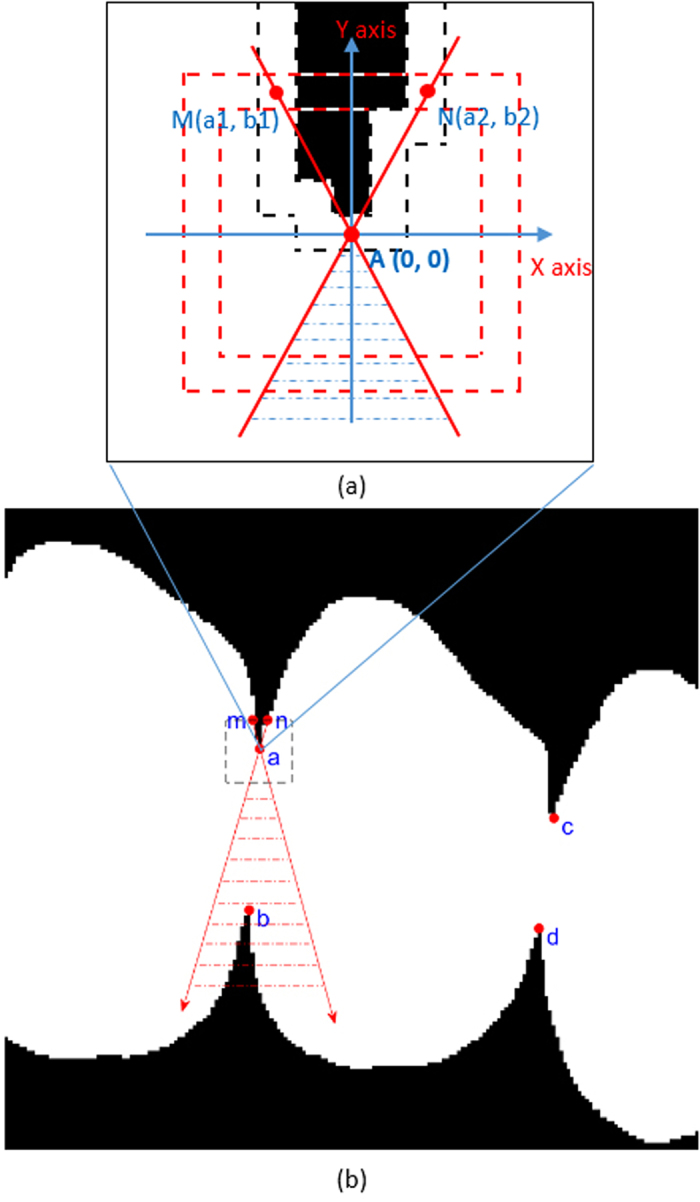
Schematic diagram of inflection point matching.

Concave point matching is the hardest step in the separation of touching rice grains. Repeated experiments showed that the number of grains touching with each other was less than five. Two grains touching together produced two concave points, meaning that these two grains could be separated using a threshold segmentation method by connecting the two concave points. The condition is more complicated for separating two or more grains which touch each other because four, six, or even more concave points are then observed, causing confusion in the inflection point matching. Therefore, collaborative constraint conditions were set based on the ECMP concave matching detection method to allow for correct matching of multiple concave points. The steps are as follows:

1. Set random point A as the basic point (BP) for concave matching and establish a coordinate system with BP as the coordinate origin. As indicated in Fig. 5(a,b), crossover points M (a1, b1) and N (a2, b2) of the grain edge (black dotted bordered) with a template of ECMP (red dotted bordered) are identified. Connect lines MA and NA, and the matching point (MP) should be located within the dashed area (blue dotted bordered) limited by MA and NA. Let f(x) represents the area outlined by the dashed lines, and f(x) can be expressed as:
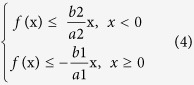
2. If two or more MPs exist within the area outlined by the dashed line, then the MP corresponding to shortest distance to BP is the true MP;
3. If no MP exists within the area outlined by the dashed line, then repeat steps 1 and 2 with the next concave point until all the concave point matching is completed.

Upon completion of matching all concave points, connect the matched concave points pairwise followed by separation of grains using self-adaptive threshold segmentation^20^.

### Calculation of grain length

In order to judge whether milled rice is head rice, ten grains of head rice of each variety were selected, and the average grain length was calculated as a reference. Then, the grain length of separated grains was calculated to compare with the reference value to decide whether the grain should be categorized as head rice.

A minimum enclosing rectangle (MER)^25^ was applied to calculate the length of the separated grains following the basic principle: Rotate the target outline for 90 degrees along certain angle (for example, 3 degrees), during which a horizontally placed MER was used to fit the target outline. The length of the MER when the minimum external rectangular area was detected after rotation of a certain degree corresponded to the length of the target rice. Then, the actual length of the rice was calculated based on the scaling of the image to the grain.

Regarding rotation of image, rotation around the origin (0, 0) is discussed first. (*x*_0_, *y*_0_) was the coordinate before rotation, and (*x*_1_, *y*_1_) was the coordinate after rotation. The rotation equation is:


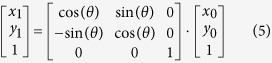


If the rotation was around point (a, b), then first move the coordinate to (a, b) before rotating the image, and finally move to the coordinate of the new origin; point (c, d) is the center after rotation:


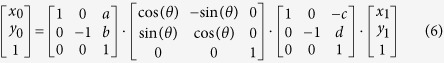


### Calculation of head rice yield

According to the Chinese national standard, head rice yield is defined as the mass ratio of head rice to the quality of net rice samples. The difference in the density and thickness of grains of same variety can be neglected. As m = ρ·V and V = S·h, the quality of grains can be estimated from the area of the grain image. In two-dimensional images, the pixels of the foreground grains and background were set to 1 and 0, respectively. The area of the total sampled grains equaled the sum of connected pixels representing the foreground grains, and the sum of pixels representing kernels that are three-fourths of the original kernel length equaled the area of the head rice. The equation for of head rice yield is:


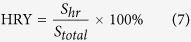


where S_*hr*_ is the sum of the touching or touching pixels of head rice, and *S*_total_ is the sum of the touching or touching pixels of all the grains.

## Results and Discussion

### Results of grain separation

**Figure 6:**
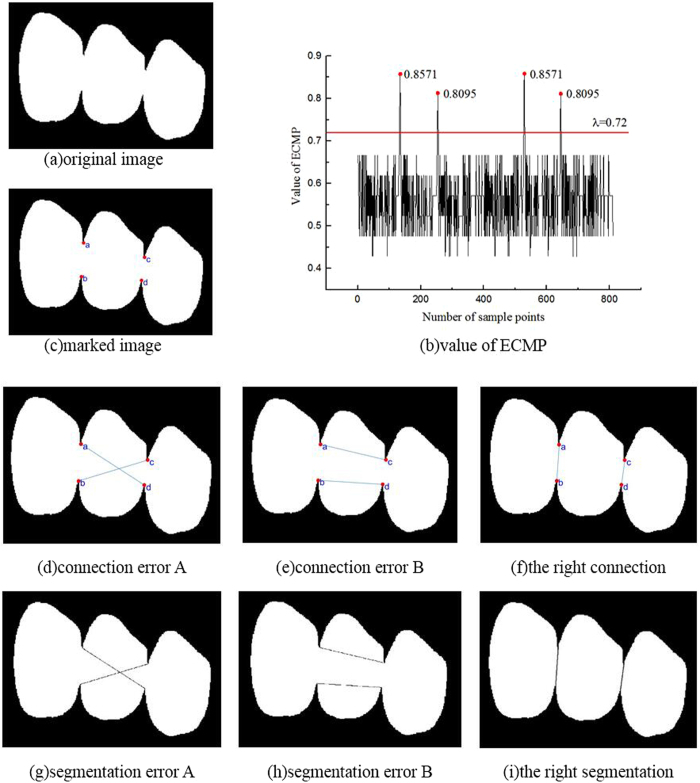
Separation of grains with ECMP method.

Figure 6(a) shows the condition in which three grains touch each other. The edge pixel with the smallest coordinate value was selected as the starting point, from which the ECMP of each edge pixel was caculated according to clockwise direction. Finally, the curve graph was drawn as shown in Fig. 6(b). The separation threshold λ = 0.72 was calculated using eqs (2) and (3), and information about four concave points was obtained. The real concave point can be pinpointed based on the peak value of the curve graph of each of the concave points (Fig. 6(c)). With a single or no constraint, concave point matching can be questionable. Figure 6(d,e,g,h) showed two possible incorrect concave point matching and separation without constraints. The concave point mismatching was solved by setting collaborative constraint conditions, and the results are presented as Fig. 6(f,i). Therefore, the pre-condition of the collaborative constraint is of critical significance for correct concave point matching.

**Figure 7:**
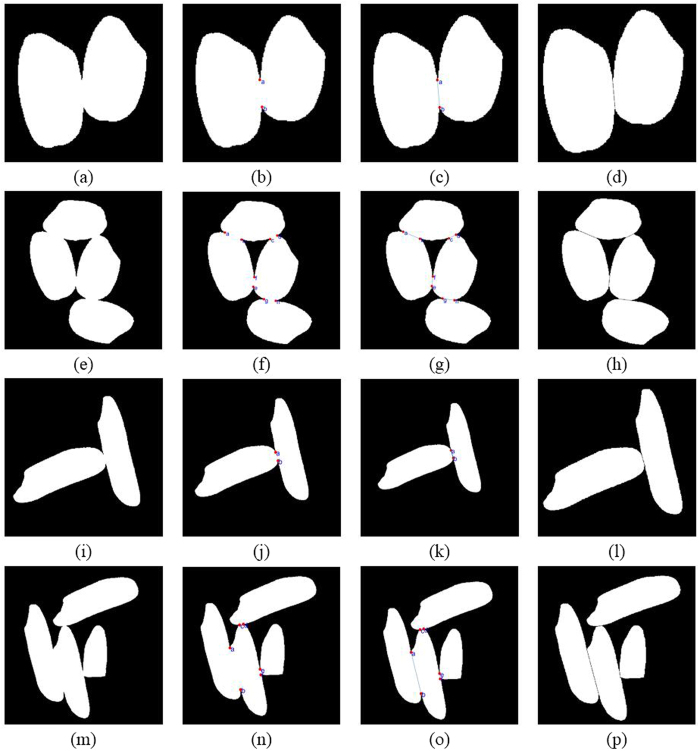
Separation results of different grain types or with different connection conditions. Note: (**a**–**d**) separation results of simple connection of japonica rice; (**e**–**h**) separation results of complex connection of japonica rice; (**i**–**l**) separation results of simple connection of indica rice; (**m**–**p**) separation results of complex connection of indica rice.

In order to explore the applicability of this research method, indica rice and japonica rice were tested, and the solution was validated with grains with no touching or significant touching. As indicated in Fig. 7, the method was independent of the grain type and was effective for both testing conditions.

The accuracy rate of the ECMP separation method used in this study was analyzed by controlling the grain numbers per image (100, 150 or 200 grains/image) and the number of acquired images. Results are shown in Table 1. With the same number of acquired images, the accuracy rate of separation with japonica rice was higher than that of indica rice, and the accuracy rate of both varieties decreased with increasing number of grains in each image. The accuracy rate with an average number of 200 grains per image decreased 2.71% compared with that of 100 grains per image. The separation accuracy for japonica rice decreased by up to 0.57% with increasing number of acquired images and grain number per image remained, which provided no significant impact on separation results, indicating that the method applied here was suitable for batch processing of rice images.

### Identification of head rice

The key in head rice identification is to calculate the grain length, which can be realized by multiple methods. The most commonly used methods include the Maximum Euclidean Distance method (MED), the central rotatable method (CR), and the MER^26^,^27^. We compared the three methods for the omission rate of head rice, false negative rate, and the amount of time consumed, and the results are presented in Table 2. The omission rate of head rice per MED and CR were both 0%. The longest grain length calculated using the two algorithms was called the theoretical grain length. However, as the actual grain length needed was slightly shorter than theoretical value, the two algorithms were not accurate enough. The MER algorithm has a higher accuracy compared with the MED as well as CR and low false detection and omission rates of less than 5%. However, the calculation time of MER was relatively longer. Increased calculation efficiency is expected while maintaining accuracy in a future study.

### Accuracy and efficiency of head rice rate

**Figure 8:**
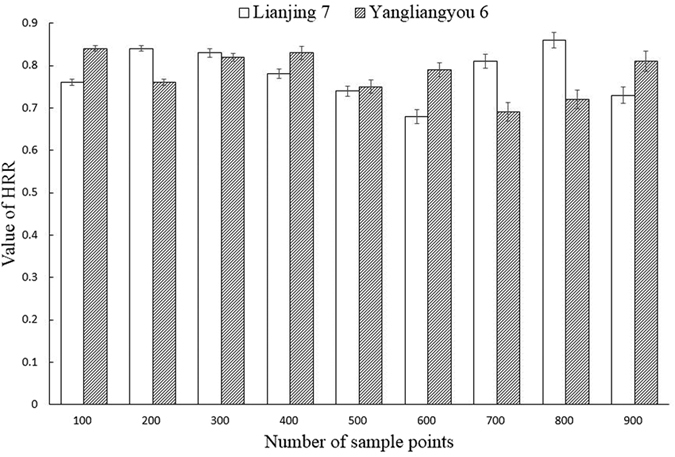
Accuracy analysis of head rice rate.

**Figure 9:**
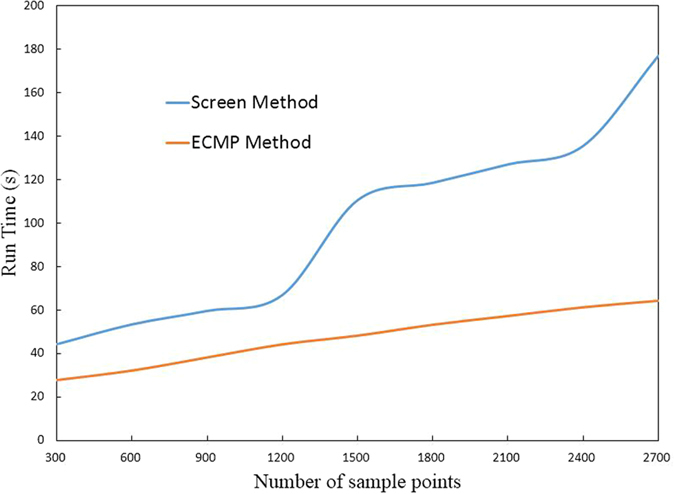
Efficiency analysis of head rice rate.

Previous studies mainly focused on grain separation^10^,^17^,^18^; systematic detection of head rice grain has not yet been supported. In the present study, image acquisition equipment was firstly enhanced under the premise of accurate grain separation in order to realize more a convenient and time-efficient image acquisition. More efficient detection method for ECMP concave point matching and collaborative constraint matching based separation was proposed as well. Finally, the advantage and disadvantage of grain length calculation algorithms were compared, and the MER-based algorithm determined to be the best. Overall experimental results of head rice grain detection are listed in Fig. 8, which shows that the accuracy of both indica and japonica rice varieties somehow decreased with increasing number of grains. Since the limited decrease degree with average relative deviation was within 3.21%. As shown in Fig. 9, running time increased gradually with the grain number increase. Advantage of this new method is not significant with sample size. However, massive of time could be saved when dealing with larger number of grains. The method was confirmed to be applicable and provides higher efficiency while increasing accuracy.

### Accuracy of equipment

At present, the image acquisition equipment is complex and diverse, which includes a mixed equipment integrating scanner^10^,^28^,^29^, video camera^30^, digital camera^7^, and other equipment. There is need to study more about how to use the equipment capable of identifying head rice in order to realize intelligent acquisition of rice images and batch acquisition. Sampling grains were placed into a vibrator that shook with a frequency of 20 Hz, making the grains evenly fall onto the conveyor belt. The time of a grain spend on the conveyor belt was 5 s. Automatic image acquisition by a digital camera was performed every 5 s, and the photos were sent to computer for processing. The number of grains per image ranged from 100 to 200, and the average acquisition rate was approximately 1,800 grains/min. A self-timer on the digital camera was combined with the characteristics of the conveyor belt and the vibrator, which satisfactorily solved the intelligent and batch acquisition of grain images.

## Conclusions

The head rice calculation currently used was improved both with equipment and a grain separation method. The performance of different methods used to calculate grain length was compared. The results showed that the image acquisition equipment used in the present study was more accurate, faster, and capable of accurate, fast, and batch image acquisition compared with other equipments. The separation method based on ECMP concave point matching and collaborative constraint matching separation was featured with high accuracy. It was capable of batch processing and has wide applicability. Among the three methods used to calculate grain length, the MER-based method had the highest accuracy, and its omission and false detection rates were lower than 5%. However, it was more time-consuming, which should be further improved. The method presented in this article was found to have high accuracy for the different rice varieties tested with a relative error between tested and actual values less than 3%, suggesting that it can be regarded as an effective detection method for head rice. This new methodology is beneficial for the improvement of head rice rate detection and could be of extensive use all over the world.

## Additional Information

**How to cite this article**: Yao, Y. *et al*. Head rice rate measurement based on concave point matching. *Sci. Rep.* **7**, 41353; doi: 10.1038/srep41353 (2017).

**Publisher's note:** Springer Nature remains neutral with regard to jurisdictional claims in published maps and institutional affiliations.

## Figures and Tables

**Table 1:** Accuracy analysis of ECMP-based separation.

Variety	Number	Number of pictures	Actual number of rice grains	Detection number by the paper	Accuracy rate
LJ 7	A11	1	104	104	100%
	A12	2	194	194	100%
	A13	3	296	295	99.66%
	Mean		99	98.83	99.83%
	A21	1	147	146	99.32%
	A22	2	308	305	99.35%
	A23	3	452	448	99.12%
	Mean		151.17	149.83	99.12%
	A31	1	196	192	97.96%
	A32	2	398	388	97.49%
	A33	3	612	596	97.39%
	Mean		201	196	97.51%
YLY 6	B11	1	96	96	100%
	B12	2	197	196	99.49%
	B13	3	308	305	99.03%
	Mean		100.17	99.5	99.33%
	B21	1	145	143	98.62%
	B22	2	312	308	98.71%
	B23	3	436	429	98.39%
	Mean		148.83	146.67	98.54%
	B31	1	210	204	97.14%
	B32	2	416	396	95.19%
	B33	3	622	601	96.62%
	Mean		208	200.17	96.23%

**Table 2:** Comparison of different methods for calculating grain length.

Variety	Number of pictures	MED			CR			MER		
		False detecting rate	Missing detecting rate	Time	False detecting rate	Missing detecting rate	Time	False detecting rate	Missing detecting rate	Time
LJ 7	1	7.14%	0.00%	3.73 s	13.33%	0.00%	1.06 s	0.00%	0.00%	8.56 s
	2	11.11%	0.00%	7.61 s	15.79%	0.00%	1.34 s	3.33%	1.76%	17.41 s
	3	13.21%	0.00%	11.18 s	16.36%	0.00%	1.61 s	4.50%	1.56%	24.33 s
**Mean**		**10**.**49%**	**0**.**00%**	**3**.**75 s**	**15**.**16%**	**0**.**00%**	**0**.**67 s**	**2**.**63%**	**1**.**11%**	**8**.**38 s**
YLY 6	1	0.00%	0.00%	3.40 s	0.00%	0.00%	1.19 s	0.00%	0.00%	7.93 s
	2	4.17%	0.00%	7.53 s	9.80%	0.00%	1.52 s	2.22%	1.29%	16.79 s
	3	7.23%	0.00%	11.39 s	10.47%	0.00%	1.81 s	2.60%	0.90%	23.48 s
**Mean**		**3**.**80%**	**0**.**00%**	**3**.**72 s**	**6**.**76%**	**0**.**00%**	**0**.**75 s**	**1**.**61%**	**0**.**73%**	**8**.**03 s**

Note: calculation time is dependent on computer configuration.
